# The Open Virtual Mirror Framework for enfacement illusions: Enhancing the sense of agency with avatars that imitate facial expressions

**DOI:** 10.3758/s13428-021-01761-9

**Published:** 2022-05-02

**Authors:** C. Martin Grewe, Tuo Liu, Andrea Hildebrandt, Stefan Zachow

**Affiliations:** 1 Computational Diagnosis and Therapy Planning Group, Department of Visual and Data-Centric Computing, Zuse Institute Berlin, Takustraße 14, 14195 Berlin, Germany; 2 Department of Psychology, Carl von Ossietzky Universität Oldenburg, Ammerländer Heerstr. 114-118, 26129 Oldenburg, Germany

**Keywords:** Enfacement illusions, Sense of agency, Virtual mirrors, Avatar faces, Real-time manipulation and morphing, Responsive facial stimuli

## Abstract

Enfacement illusions are traditionally elicited by visuo-tactile stimulation, but more active paradigms become possible through the usage of virtual reality techniques. For instance, virtual mirrors have been recently proposed to induce enfacement by visuo-motor stimulation. In a virtual mirror experiment, participants interact with an avatar that imitates their facial movements. The active control over the avatar greatly enhances the sense of agency, which is an important ingredient for successful enfacement illusion induction. Due to technological challenges, most virtual mirrors so far were limited to the imitation of the participant’s head pose, i.e., its location and rotation. However, stronger experiences of agency can be expected by an increase in the avatar’s mimicking abilities. We here present a new open-source framework for virtual mirror experiments, which we call the Open Virtual Mirror Framework (OVMF). The OVMF can track and imitate a large range of facial movements, including pose and expressions. It has been designed to run on standard computer hardware and easily interfaces with existing toolboxes for psychological experimentation, while satisfying the requirement of a tightly controlled experimental setup. Further, it is designed to enable convenient extension of its core functionality such that it can be flexibly adjusted to many different experimental paradigms. We demonstrate the usage of the OVMF and experimentally validate its ability to elicit experiences of agency over an avatar, concluding that the OVMF can serve as a reference for future experiments and that it provides high potential to stimulate new directions in enfacement research and beyond.

## Introduction

Since the discovery of the rubber hand illusion in 1998 by Botvinick and Cohen (Botvinick & Cohen, 1998), research on the plasticity of self-representations is progressing rapidly (Cowie, Sterling, & Bremner, 2016). In those early experiments demonstrating the illusion, the hand of an individual was hidden under a table while the person was looking at a physical replica placed on top of the own hand. An experimenter stroked both the real hand and the replica, simultaneously with a brush. The observation was that individuals experienced stronger ownership over the replica as compared with a condition in which the stroking of the participants’ real hand was delayed. In follow-up studies, it has been further demonstrated that a synchronous *visuo-tactile* stimulation also led to similar illusions for other body parts. They are often referred to as embodiment illusions (cf. comprehensive reviews (Kilteni, Maselli, Kording, & Slater, 2015; Braun et al., 2018; Porciello, Bufalari, Minio-Paluello, Di Pace, & Aglioti, 2018)). The so-called enfacement illusion also belongs to these phenomena of increased ownership, which occur as a consequence of synchronous visuo-tactile stimulation, i.e., when a participant’s face is stroked in synchrony with another face (Tajadura-Jiménez, Longo, Coleman, & Tsakiris, 2012; Tajadura-Jiménez, Grehl, & Tsakiris, 2012; Minio-Paluello, Porciello, Gandolfo, Boukarras, & Aglioti, 2020).

In everyday life, the sense of ownership (SoO) is typically co-experienced with the sense of agency (SoA). For instance, individuals experience both senses simultaneously for their legs when they walk. The SoO describes the feeling that the legs belong to one’s own body and the SoA refers to the experience of an active control over their movements. While the exact mechanism of the interplay between these two senses is still not entirely understood, both are apparently of crucial importance for own body representations (Braun et al., 2018). However, many experiments so far only applied visuo-tactile stimulation, which is a passive experience for a studied individual and, as such, it is primarily linked to the SoO. To strengthen the experience of the SoA in experimental setups, attempts have been made to establish more active paradigms. One such approach is the *visuo-motor* stimulation, where the replica can mirror movements of a studied individual.

For instance, visuo-motor stimulation was achieved in rubber hand experiments by mechanically connecting the participant’s hand to the replica with a rope such that movements were directly transferred. However, the degrees of freedom of the movements that could be transferred by this technique were overly limited, e.g., to the imitation of the participant’s hand pose (Dummer, Picot-Annand, Neal, & Moore, 2009), finger tapping (Kalckert & Ehrsson, 2014a), or button pressing (Braun, Thorne, Hildebrandt, & Debener, 2014). More recently, Caspar and colleagues (Caspar et al., 2015) published a do-it-yourself robotic hand that can mimic a larger range of actions. They connected a self-made sensor glove with a 3D printed robotic hand using a micro-controller. Their novel technology has been demonstrated to induce higher SoA in active vs. passive and synchronous vs. asynchronous conditions. It can be easily programmed to achieve a large variety of experimental conditions.

In contrast to the robotic hand where motion can be convincingly displayed by an articulated model, the creation of an active facial replica for the study of the enfacement illusion is comparably more difficult. Facial motion is driven by the joint action of many muscles and becomes visible by complex deformation of the facial surface. Furthermore, humans are particularly sensitive to even subtle artifacts perceived in faces which can cause disturbing effects such as uncanny valley phenomena (de Borst & de Gelder, 2015; Kätsyri, Förger, Mäkäräinen, & Takala, 2015). The implementation of convincing facial motion in robotic faces can still be regarded as a problem that is not well resolved yet.

Fortunately, virtual faces provide an alternative to physical replicas. For instance, an avatar can imitate the participant’s facial motion in a virtual mirror. Various entertainment productions have demonstrated that realistic facial animation in avatars is possible. Markerless face tracking has greatly advanced in the last decade (Wu & Ji, 2018), and recent work indicates that it might even become possible with head mounted displays in the near future (Lombardi, Saragih, Simon, & Sheikh, 2018; Thies, Zollhöfer, Stamminger, Theobalt, & Nießner, 2018). Further, the usage of virtual faces provides theoretical and methodological advantages (Braun et al., 2018; Porciello et al., 2018). In fact, enfacement illusions induced by visuo-motor stimulation were already replicated in different virtual mirror experiments (Serino et al., 2015; Estudillo & Bindemann, 2016; Ma, Sellaro, & Hommel, 2018). However, in these implementations, the imitation of movements by the avatar was largely limited, e.g., to the participant’s gaze or head pose, i.e., its location and orientation (Porciello et al., 2018). The tracking of facial expressions was neglected even if it is indicated that synchronized expressions can enhance the SoA (Kokkinara & McDonnell, 2015; Gonzalez-Franco, Steed, Hoogendyk, & Ofek, 2020).

Following the efforts of Caspar et al. (Caspar et al., 2015) in the realm of the rubber hand illusion, our goal was to create and validate a virtual mirror framework for enfacement experiments that (a) provides visuo-motor stimulation by realistic imitation of a large range of facial movements including pose and expressions, (b) can be easily employed in a typical lab environment, (c) conveniently interfaces with well-established software for psychological experimentation, and (d) fosters sustainability, extensibility, and replicability in enfacement research. In the first part of this paper, we discuss existing virtual mirror implementations and related virtual reality (VR) techniques. We then describe the Open Virtual Mirror Framework (OVMF), its usage, and extension for new experiments. The source code of the OVMF is publicly available^1^. We validate the OVMF in an experimental study by investigating if synchronized facial expressions contribute to inducing stronger SoA as compared to avatars imitating head pose only. Finally, we discuss the outcomes of the validation study and point out new opportunities that arise from using the OVMF for enfacement studies and further psychological experiments.

## Available virtual mirrors for enfacement research

The non-verbal behavior in human faces basically comprises eye gaze, head pose, and facial expressions. In principle, all of these movements are also perceivable in one’s own face when one looks into the mirror. As discussed by Porciello and colleagues (Porciello et al., 2018), difficulties arise if only eye gaze is used to create synchronous visuo-motor stimulation such as in (Estudillo & Bindemann, 2016). The reason for this is that individuals instructed to gaze away from the avatar’s eyes naturally also shift away their focus, such that they lack the opportunity to perceive the imitation of gaze shift carried out by the avatar. In contrast, changes in location and orientation of the head are more obviously traceable by a participant and the focus of attention can remain on the avatar. Therefore, almost all other available virtual mirror experiments so far induced enfacement illusions with avatars that can imitate the participant’s head pose (Serino et al., 2015; Ma, Sellaro, Lippelt, & Hommel, 2016; Ma et al., 2018; Zhang, Hommel, & Ma, 2021).

To the best of our knowledge, only a single attempt has been made to investigate how the imitation of facial expressions effects the SoA (Kokkinara & McDonnell, 2015). In the respective study, the authors demonstrated that individuals tend to experience a high SoA over avatars that imitate their facial expressions. These results are in line with the work of Gonzalez and colleges (Gonzalez-Franco et al., 2020). Although their method was not conceived to directly mirror the facial expressions of participants, they animated the avatar’s verbal and non-verbal behavior based on real-time analysis of the participants’ speech. They found that the strength of the enfacement illusion was positively associated with the strength of the facial behavior displayed by the avatar.

The above-summarized studies suggest that avatars which can mimic a large range of facial behaviors are beneficial for virtual mirror experiments, but their direct contribution to increase the SoA over pose-only virtual mirror solutions remains to be demonstrated. One reason why we still lack such a demonstration is the importance of a high temporal and semantic congruence that needs to be achieved by the imitating avatar (Kilteni et al., 2015). In other words, robust enfacement illusions require real-time tracking and transfer of participants’ movements as well as a high photographic and behavioral realism of the avatars. These requirements, however, render the implementation of virtual mirror experiments technically highly challenging.

### Temporal congruence

Temporal congruence essentially refers to the delay that is introduced by the virtual mirror system between the movement of a participant and the appearance of the corresponding action of an avatar displayed on the screen. An intentional delay in the order of seconds is usually added in an asynchronous control condition to diminish the SoA (Porciello et al., 2018). In a synchronous condition, participants’ movements are to be transferred without delay, such that the experience of agency is maximized.

For technical reasons, however, a certain latency of the digital system is unavoidable. In such a system, all delays caused by the sensor measurements, computation time, or the response rate of a display will sum up. We refer to this as the end-to-end latency, which can be determined by a video-based measurement technique (He, Liu, Pape, Dawe, & Sandin, 2000). Even in simple experimental setups, the end-to-end latency quickly exceeds 80 ms (Shimada, Fukuda, & Hiraki, 2009; Ismail & Shimada, 2016), and likely increases for more sophisticated VR systems. The effect of latency on the SoA was investigated using experimental setups that were precisely calibrated. Despite some controversy, researchers generally agree that a critical range of 200–300 ms exists (Shimada et al., 2009; Ismail & Shimada, 2016; Waltemate et al., 2016; Wen, 2019). Delays greater than this range cause a decrease of SoA, while delays below this value barely have any effect.

Today, sophisticated virtual mirror systems with professional tracking, rendering, and display devices can achieve a latency of about 60 ms (Waltemate, Hülsmann, Pfeiffer, Kopp, & Botsch, 2015; Latoschik et al., 2017; Waltemate, Gall, Roth, Botsch, & Latoschik, 2018). Cheaper setups that are affordable for typical research labs become eligible by the usage of standard computer hardware. Latoschik and colleagues (Latoschik, Lugrin, & Roth, 2016) demonstrated that a virtual mirror for embodiment research can reach an end-to-end latency of about 150–200 ms, which is below the critical range. Their system can also track the facial expressions of study participants, but it is based on proprietary components and is not publicly available. One virtual mirror system was specifically designed and released for enfacement research (Ma et al., 2016). The authors reported that it was able to imitate the participant’s head pose with a very small latency of 40 ms. Unfortunately, they did not report details on the measurement method, but in the light of the performance of the other systems mentioned above, the latency was unlikely determined end-to-end.

### Semantic congruence

According to the review by Kilteni and colleges (Kilteni et al., 2015), semantic congruence pertains to higher-order features of an avatar, e.g., to which extent it resembles the static and dynamic shape, anatomy, and structure of a physical human body. Often, it is also termed anthropomorphism or human-likeness. In case of faces, two aspects are particularly important: Photographic realism (e.g., the level of detail of shape and texture or the naturalness of shading) and behavioral realism (e.g., similarity of the movements to a human face).

Various methods are available today which allow the construction of avatars with photorealistic facial shape and texture from 3D scans, videos, or photographs (Ichim, Bouaziz, & Pauly, 2015; Grewe & Zachow, 2016; Achenbach, Waltemate, Latoschik, & Botsch, 2017; Grewe, Le Roux, Pilz, & Zachow, 2018). The benefit of using realistic shapes and textures has been demonstrated for the rubber hand (Lin & Jörg, 2016), the embodiment (Latoschik et al., 2017; Zibrek & McDonnell, 2019), as well as the enfacement illusion (Gorisse, Christmann, Houzangbe, & Richir, 2019). However, studies often reported undesired effects of photorealistic avatars as well. Such effects can almost always be attributed to the uncanny valley phenomenon (de Borst & de Gelder, 2015; Kätsyri et al., 2015).

Amongst others, the uncanny valley is related to a perceptual mismatch, e.g., an imbalance of an avatar’s photographic and behavioral realism, which causes a disturbance in the visual processing of the face. It often occurs when photorealistic faces are animated (Dobs, Bülthoff, & Schultz, 2018). Avatars which were created with well-established software tools such as FaceGen^2^, MB-Lab^3^, FACSHuman^4^, or FaReT^5^ contain facial expressions that were designed by artistic experts (Gilbert, Demarchi, & Urdapilleta, 2018; Hays, Wong, & Soto, 2020). The design of realistic (dynamic) facial expression is particularly challenging, and artifacts or implausibilities can easily occur (Lewis et al., 2014). Consequently, a perceptual mismatch might result if they are combined with highly photorealistic facial shapes and textures. As demonstrated recently, less mismatch and higher perceived congruence can be achieved if facial expressions are statistically learned from a large 3D facial expression database (Grewe, Liu, Kahl, Andrea, & Zachow, 2021).

### Practical aspects

Not only technical feasibility but also practical implementation is important for the usage of an experimental method in a behavior study laboratory. If a virtual mirror is affordable, easy to use, and extensible, more laboratories might use it. This increases the reproducibility and replicability of psychological findings in the domain of the enfacement illusion. Some software libraries were publicly released which could, in principle, be used for a virtual mirror implementation (van der Struijk, Huang, Mirzaei, & Nishida, 2018; Garolera, Llorach, Agenjo, & Blat, 2019). However, their setup and usage are elaborate and extensions would require advanced programming skills.

In enfacement experiments, the exposure of participants to the virtual mirror is usually followed by the acquisition of responses to some questionnaire items or behavioral tasks. Digital capture of such responses is arguably of advantage, and it is usually implemented with toolboxes that were specifically designed for psychology and behavioral sciences. An interface to the virtual mirror, which allows its integration into such toolboxes like PsychoPy^6^ or OpenSesame^7^, is the most convenient solution, but is, to our knowledge, not provided by any previously published methodological frameworks.

## The open virtual mirror framework

**Fig. 1:**
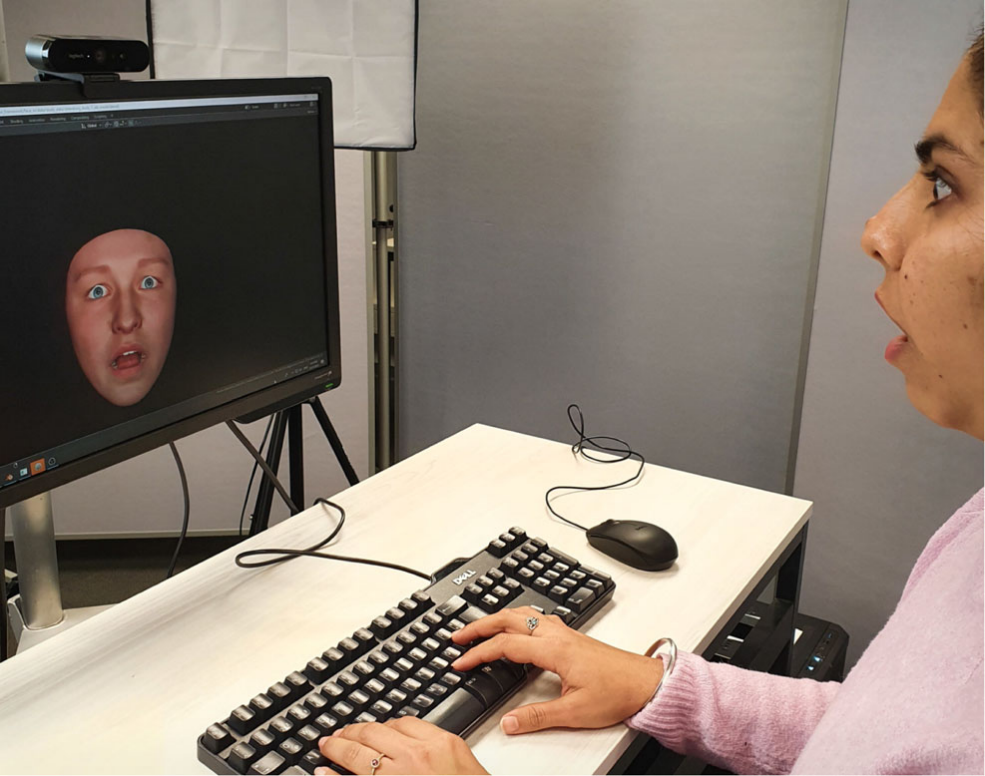
A typical setup of a virtual mirror experiment with the OVMF

In line with the requirements of enfacement research and challenges that occurred in previous implementations, we developed a new open-source framework for virtual mirror experiments that we call the Open Virtual Mirror Framework (OVMF). As illustrated in Fig. 1, the OVMF runs on using standard computer hardware with a webcam mounted to the top of the screen. It tracks the facial movements of the participant without markers and synchronously transfers them to an avatar which is rendered and displayed with a latency fairly below 200 ms. The Windows and Linux operating systems are currently supported, and a porting to Mac should be easily possible.

In this section, we describe the OVMF in a top-down manner. We first exemplify how a virtual mirror implemented with the OVMF can be embedded into a PsychoPy experiment. Then, we provide details about the architecture of the OVMF, its major components, and how it can be extended. Finally, we demonstrate a method for convenient latency calibration of a virtual mirror that is newly installed on a lab computer.

### Usage of the OVMF in enfacement experiments

**Fig. 2:**
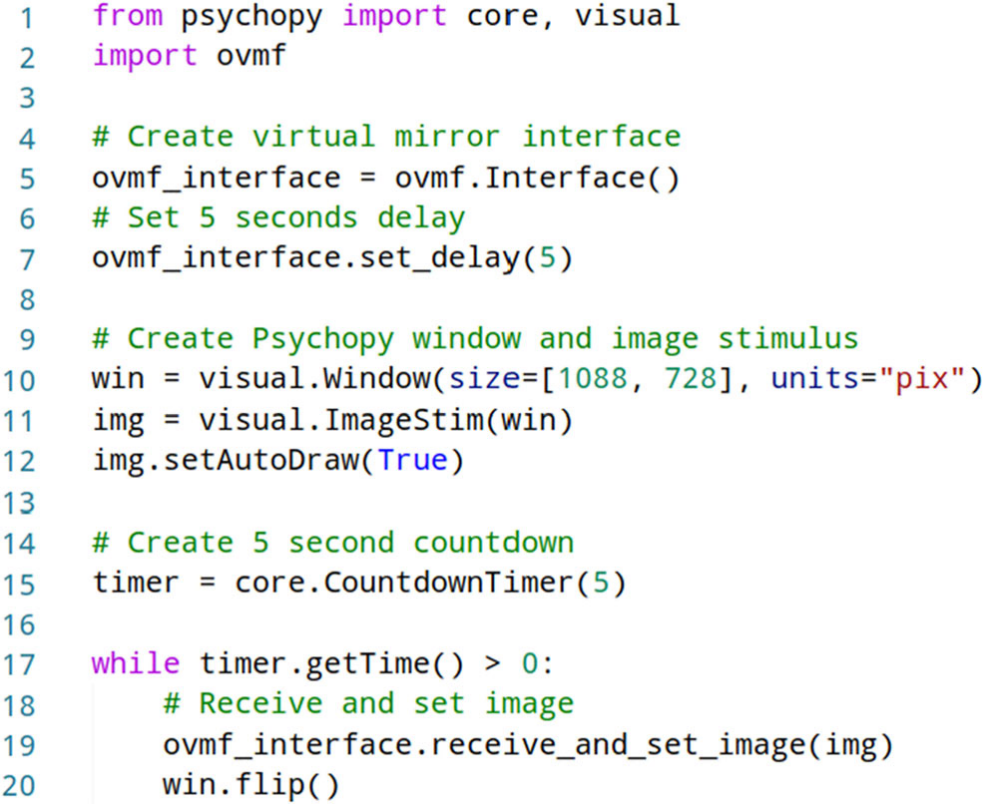
Source code for an elementary virtual mirror experiment implemented in PsychoPy, which uses the OVMF framework

The OVMF provides an interface such that it seamlessly inte grates with other Python toolboxes. This means the framework can be entirely controlled from a scripted experiment. Initially, the OVMF is launched in the background before the actual experiment is started. Figure 2 shows the source code of a PsychoPy experiment, which integrates the virtual mirror. After the interface is created (line 5) and the delay parameter is adjusted (line 7), the PsychoPy window and an image stimulus are created (line 10 to 12).

Then, the virtual mirror is shown for 5 s by repeated retrieval and a new image of the rendered avatar as specified in lines 15 to 20 is displayed. After the virtual mirror phase, the experiment continues as desired, e.g., with the assessment of a questionnaire to capture reports of the enfacement illusion. Similar to the adjustment of the delay in line 6, the interface provides a set of convenience functions, which allow dynamic adjustment of the OVMF during the experiment, including switching of avatar identities or amplification of imitated expressions.

### Architecture of the OVMF

**Fig. 3:**
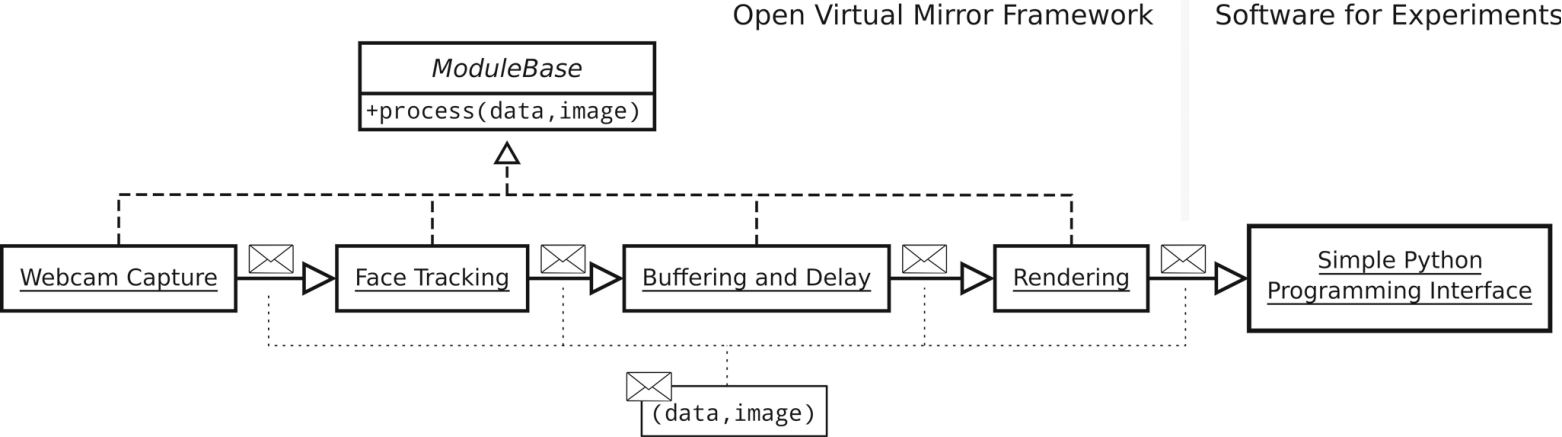
The pipeline of a virtual mirror usually contains an initial module that captures camera images. A message with the new image and associated data is then fed into the pipeline. This is processed by the tracking module, which adds the detected facial pose and expressions to the message. After a user-defined delay, the tracking parameters are transferred to the avatar which is rendered accordingly. Finally, the rendered image can be received and displayed through the Python interface in the experiment’s graphical window. Basic functionality such as the message-passing mechanism is encapsulated in a base module

The OVMF is implemented in Python and divided into multiple modules. Figure 3 shows a schematic overview of the modules, which are contained in a typical virtual mirror. The modules are arranged in a pipeline and messages are passed from the initial to the last module in a feed-forward fashion.

A message consists of an image and the data associated with it. The data is stored in a Python dictionary to provide easy access. Image and data are processed and/or manipulated by the modules in the pipeline. For instance, a new image of the participant’s face is captured by the webcam and passed to the face-tracking module. The tracker determines the facial pose and expressions and adds them to the data dictionary before the message is fed forward.

The modules operate in parallel so that different messages can be processed simultaneously. For instance, face tracking can be performed for a new webcam image while a previous frame is still being processed for the animation and rendering of the avatar.

The pipeline specifies the behavior of the OVMF. By re-arrangement and extension of the pipeline, other behaviors for different experimental conditions can be implemented. For instance, the buffer and delay module can be removed if asynchronous visuo-motor stimulation is not needed, while a module can be added that amplifies the tracked expressions. Each pipeline is defined in a text file that can be specified during the launch of the OVMF.

### Basic modules for a virtual mirror experiment

The OVMF currently provides a set of modules which are needed for basic virtual mirror experiments. We briefly describe them here.

#### Webcam capture

One of the main components in a virtual mirror is the camera, which captures a participant’s face. The camera is accessed via the OpenCV^8^ Python library, which provides an abstraction layer for a wide variety of devices and platforms. The module continuously requests a new image from the camera and determines the time of image acquisition. It also estimates the camera’s intrinsic parameters, which are important for transformations from camera to screen space. The image and intrinsics are added to the data dictionary and fed into the pipeline.

#### Face and expression tracking

The markerless tracking of facial pose and expressions is achieved using the OpenFace^9^ library (Baltrusaitis, Zadeh, Lim, & Morency, 2018). The provided methods have been trained on a large and diverse database including in-the-wild images. A good performance can thus be expected for a wide range of participants and conditions in the lab. The library estimates 3D pose as well as presence and intensity of several Action Units (AUs) (Ekman, Friesen, & Hager, 2002). Since OpenFace is implemented in C++, we created a wrapper that exposes its main functionality as a Python module. As such, the framework can handle the tracker similarly to any other module, which greatly enhances usability and extensibility as compared to previous frameworks (van der Struijk et al., 2018). After successful processing of an image, pose and AU estimates are added to the data dictionary.

#### Avatar animation and rendering

**Fig. 4:**
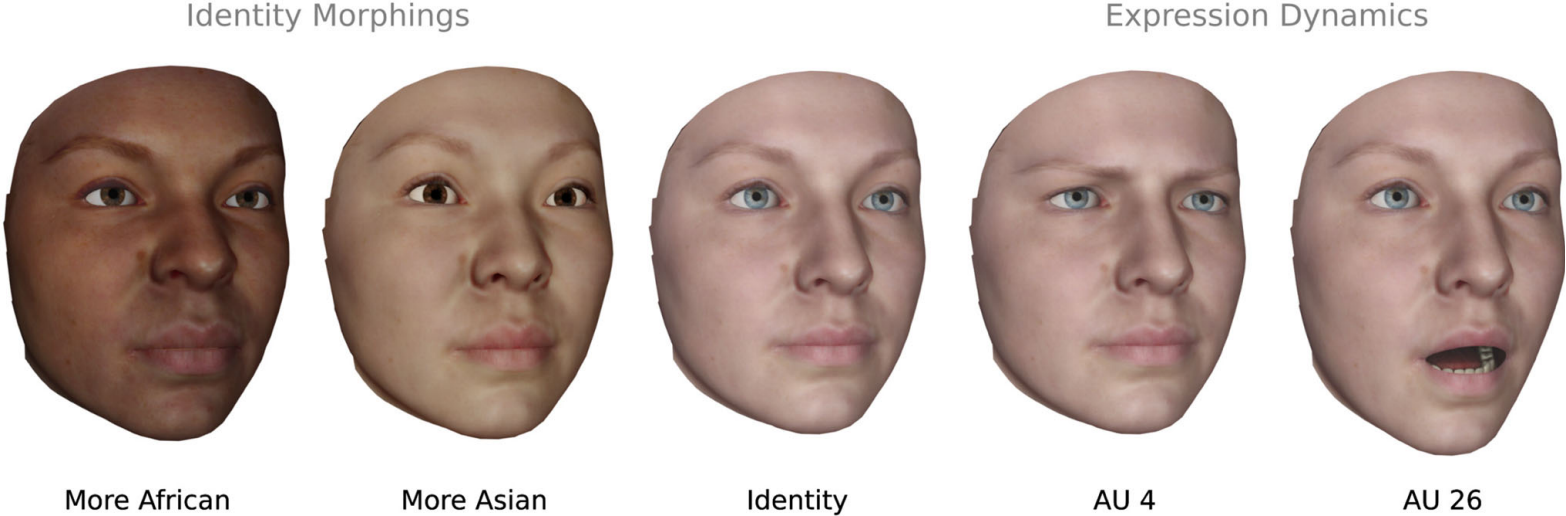
An individualized avatar (*middle*) created by the FexMM using the methods described in (Grewe et al., 2021). The model’s parameters allow intuitive and plausible manipulation of facial identity (*left*) and expressions (*right*)

As discussed above, enfacement illusions depend on the semantic congruence of an avatar, i.e., its photographic and behavioral realism. The avatars provided with the OVMF were therefore created with a statistical face model, called the Facial Expression Morphable Model (FexMM) (Grewe et al., 2021). FexMM avatars were shown to provide a high photographic and behavioral realism. Further, these avatars can be individualized in facial shape and texture from a few photographs of the participant which was recruited for an experiment. The FexMM also provides the ability to manipulate a large range of identity features (see Fig. 4). The OVMF comes with several readily prepared avatars of this kind.

Almost all related virtual mirror systems use proprietary real-time rendering software, like Unity^10^ or Vizard^11^ (Latoschik et al., 2016; Latoschik et al., 2017; Ma et al., 2016; Waltemate et al., 2018; van der Struijk et al., 2018). We utilize the open-source tool Blender^12^ and its render engine Eevee. Since Blender provides a comprehensive Python scripting interface, it nicely integrates into our framework. On retrieval of a new message, the rendering module transfers the new parameters to the avatar. A rendered image of the updated scene is requested and sent to subsequent modules. The rendering module currently allows switching between multiple avatar identities, but arbitrary morphings are planned for future releases.

#### Implementation of a new module

The modular design of the OVMF enhances its extensibility (see also Fig. 3). The development of new modules becomes easy by hiding any technical details. All basic functionality is encapsulated in a base class called ModuleBase and can simply be inherited when a new module is created.

**Fig. 5:**
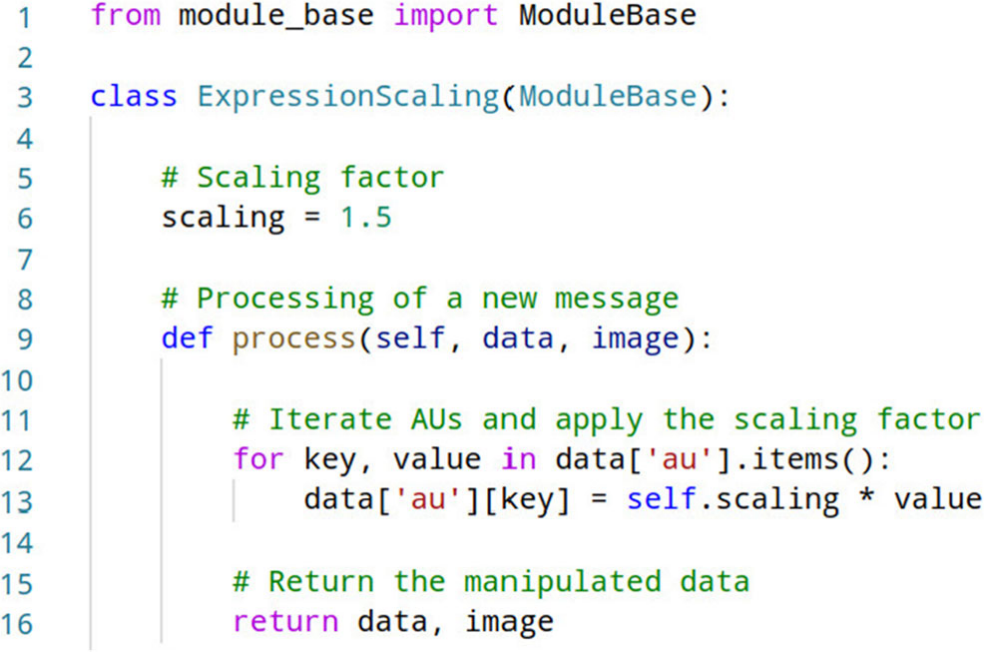
Example implementation of a OVMF module which scales the tracked AUs on-the-fly. The module receives the data dictionary and the image captured by the webcam, manipulates the AUs parameters, and returns them for forwarding to subsequent modules

For instance, one might want to investigate the effect of expression amplification on the strength of the enfacement illusion. Figure 5 shows the source code of an example module which amplifies or attenuates the tracked expression parameters by up- or down-scaling of the AUs intensities. Here, the scaling factor defined in line 6 scales the intensities by 1.5. The scaling of an incoming (data, image) tuple is achieved by implementation of the process function in lines 9 to 16. It essentially iterates the tracked AUs and applies a predetermined scaling factor.

### Latency calibration of the OVMF

The OVMF is intended to run on standard computer hardware. In general, such systems are highly heterogeneous, which also affects the end-to-end latency of a virtual mirror. For instance, the latency depends on the availability of computational resources, the intrinsic lag of the camera (sensor exposure, internal image processing), or the display’s response time. We therefore provide methods for analysis and calibration of the framework’s internal and overall latency and demonstrate their application.

#### Methods for latency measurement

The OVMF uses the PC’s monotonic clock for the measurement of time spend within the framework. This clock provides sufficient accuracy (at least in milliseconds on most operating systems) and is not affected by time adjustments. We determine the duration that a message needs to be processed by each module and within the entire pipeline, i.e., from retrieval of a captured image until flipping of PsychoPy’s backbuffer (see line 19 and 20 in 2). Although this provides insight into the distribution of the framework’s internal workload, the camera’s and monitor’s intrinsic lag are not yet considered.

The second method allows determination of the end-to-end latency, i.e., the time from the occurrence of a facial motion in front of the camera until the appearance of the imitating avatar on the screen. This can be done by simultaneously recording a video of both the real and the avatar’s face (He et al., 2000). The end-to-end latency can then be determined from the frame shift between the original and mimicked motion. We have implemented a largely automatized procedure to determine these shifts.

**Fig. 6:**
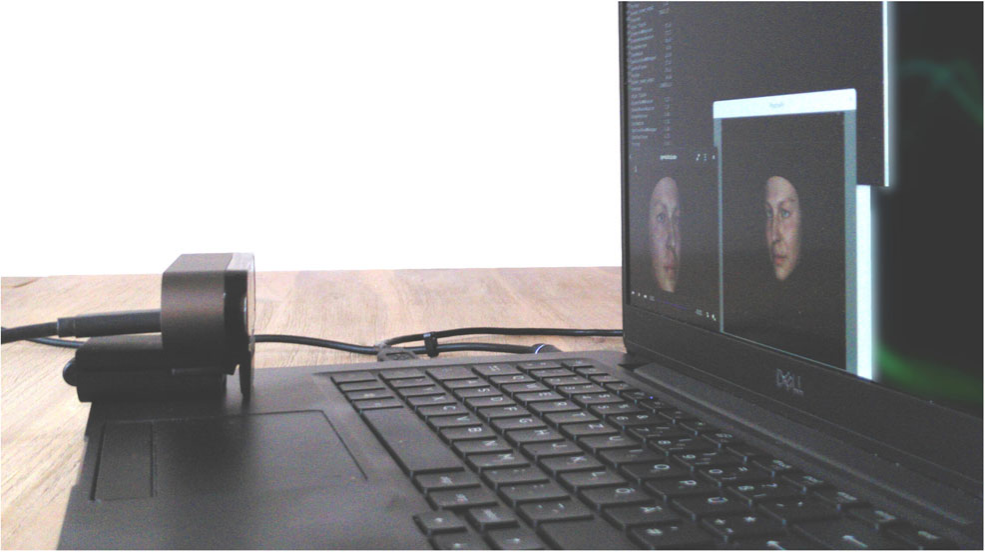
The webcam is placed in front of the video showing the animated avatar. The mimicking avatar produced by the framework is placed next to the video such that it can be easily recorded by a second camera (not shown here)

**Fig. 7:**
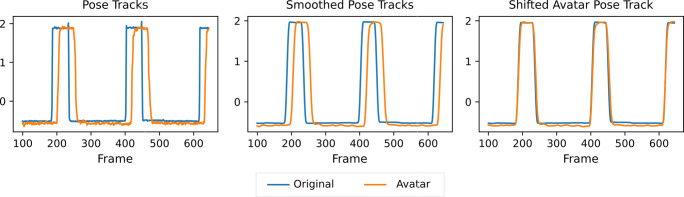
Head rotation tracked by OpenFace for the original motion (*blue*) and the mimicking avatar (*orange*). The original poses were z-transformed and smoothed to determine the frame shift. The accuracy of the frame shift is limited by the frame rate of the recorded video (here a smartphone with 120 fps), but the periodicity of the tracks due to continuous replay of the video can be exploited. For our latency analysis, we sampled *N* = 50 sub-sequences of 3 s (i.e., 1.5 times the length of the periodical motion in the synthesized video) and averaged all frame shifts

In a nutshell, we created a video that shows an avatar face performing an abrupt head rotation. Initially, it is shown frontal (hold for 1.5 s) and then turns its head 25^∘^ to the left (hold for 0.5 s). Figure 6 illustrates how the video is continuously replayed on the screen and captured by the webcam. The avatar in the virtual mirror is displayed next to the video. Both faces are recorded for 30 s by a second camera with sufficiently many frames per second (fps). The recorded video is fed into OpenFace for automatic pose tracking. As illustrated in Fig. 7, the frame shift can finally be determined by correlation analysis.

#### Experiments

We ran our experiments in Linux on a Dell Latitude 7300 laptop (Intel i7-8665U CPU with an integrated graphics card, 32 GB RAM, 60-Hz IPS display) and a Logitech BRIO webcam (USB 3.1) operating with 120 fps in 640 × 480 resolution and MJPG compression. We configured a simple pipeline containing modules for webcam capture, face tracking, and avatar rendering. The rendered image was received and displayed in a PsychoPy window similar to the script in Fig. 2. We used Blender 2.90.1, PsychoPy 3.2.4, and Python 3.8.6.

#### Results

Using the above procedure, we determined an average end-to-end latency of the virtual mirror pipeline of 160.83 ms (*SD* = 6.57 ms). The values ranged from 150 to 175 ms and thus stayed clearly below the critical range of 200–300 ms. Remarkably, an average latency of 81 ms (*SD* = 3.78 ms) was only caused by our camera and display. We also noted that the camera lag increased about 50 ms when uncompressed YUYV format was used.

**Fig. 8:**
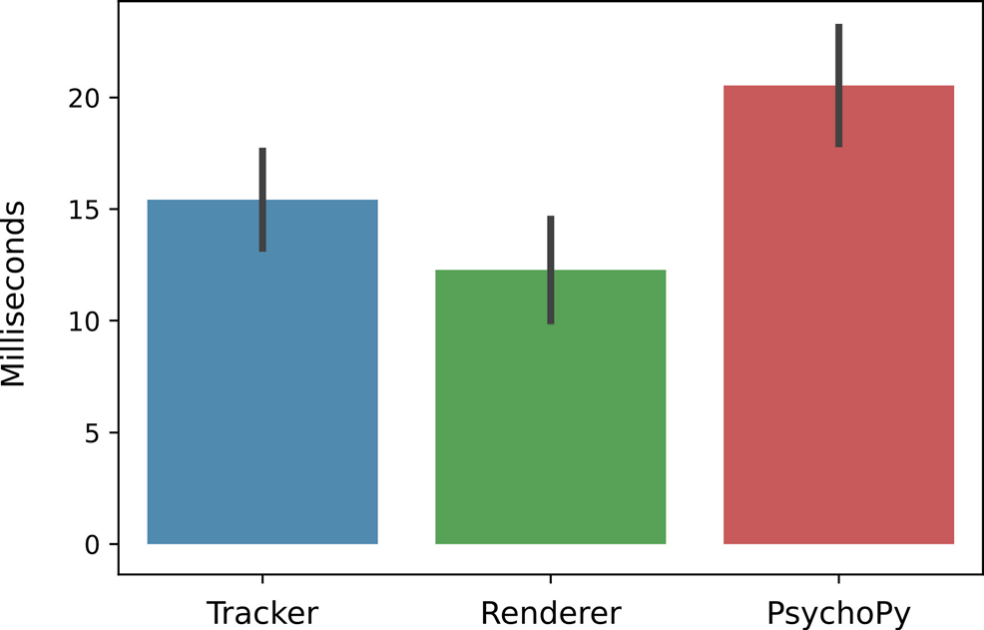
The time spent in each module’s process function within the simple pipeline

Inspection of the time spent within the OVMF revealed that the average processing time of a message was 73.46 ms (*SD* = 8.50 ms). Figure 8 indicates the latencies attributed to each module. As expected, tracking (*m* = 15.42 ms, *SD* = 2.18 ms) and rendering (*m* = 12.27 ms, *SD* = 2.27 ms) were processing-intensive tasks. At least for simple scenes like a virtual mirror, our results indicate that Blender performs similarly fast as proprietary game engines such as Unity, which needed about 8.23 ms (*SD* = 4.77 ms) in a comparable setup (van der Struijk et al., 2018). Most time was needed within PsychoPy (*m* = 20.54 ms, *SD* = 2.61 ms), which is likely due to synchronization with the displays refresh rate (60 Hz causes a flip only every 16.76 ms). In a real experiment, the synchronization with the screen (i.e., PychoPy’s waitBlanking option) can safely be disabled. This resulted in about 7-ms improvement of end-to-end latency in our case since a new image can already be retrieved concurrently. The remaining time of about 25 ms can be attributed to the framework’s processing overhead, such as the transfer of images between modules.

## An enfacement study utilizing the OVMF

We also conducted an experiment to evaluate the OVMF. The goal was to test its capability to induce the SoA over a facial avatar. In line with the previous literature, we expected an increase of the SoA by expression synchrony in addition to head pose synchrony only.

### Design

The contribution of each motion component to the enfacement illusion, i.e., pose vs. expressions, was investigated using three different experimental conditions, in which only pose (P), only expressions (E), and both pose and expressions (PE) were imitated by the avatar (see Table 1). We expected individuals to experience highest SoA after interacting with an avatar in the PE condition.

**Table 1:** The four conditions used in our evaluation study

Condition	Description
B	Continuous replay of a prerecorded rest sequence (Baseline).
P	Transfer of tracked head pose while expression remains neutral (only pose).
E	Transfer of tracked expressions while pose remains at rest (only expressions).
PE	Transfer of tracked pose and expressions (pose and expressions).

In previous enfacement experiments, a comparison between synchronous and asynchronous conditions was central to avoiding confounding effects (Riemer, Trojan, Beauchamp, & Fuchs, 2019). Due to the importance of temporal congruence, a delay has most often been added to the tracked facial motion as an asynchronous baseline (Serino et al., 2015; Ma et al., 2016). However, even large delays of a few seconds led to relatively high ratings of SoA (Farrer, Bouchereau, Jeannerod, & Franck, 2008). This might be because participants remember and recognize their movements in spite of the delay. This would bias the self-reported SoA in the asynchronous condition (Moore, 2016).

Therefore, we included a baseline condition (B), which reduces the influence of movement recognition on the experience of agency. The goal was to entirely decouple the movements of the avatar from those of the participant. We thus recorded a video sequence displaying a face at rest with neutral expression and stored the parameters which were tracked by OVMF into a file. Instead of the participant’s movements, these parameters were continuously replayed and transferred to the avatar in the baseline condition B. The transfer of subtle movements (e.g., breathing) from the prerecorded sequence has the advantage that the avatar looked more natural as compared to an entirely static face shown on the screen.

### Participants

We used G*Power 3.1.9^13^ to estimate the minimal required sample size. For *α* = .05, *β* = 0.95, and effect size of *r* = .72 provided by a previous study (Ma et al., 2018), the estimated minimal sample size to detect at least one difference between the experimental conditions was approximately *n* = 12. For robustness with respect to multiple comparisons, we did not stop recruiting participants after the minimal number was achieved, but doubled the sample size. Previous enfacement studies did not reveal substantial differences in the strength of the SoA between sexes (Tajadura-Jiménez et al., 2012). We therefore recruited only females for convenience. The final sample comprised of *N* = 24 German-speaking female Caucasian volunteers (*M*_*age*_ = 23.92,*SD*_*age*_ = 3.50).

The study was reviewed and approved by the Committee of Ethics of the German Psychological Society (DGPs, reference number: AH 082018). All individuals in the sample provided their written informed consent to participate in this study. They were remunerated with €8 per hour.

#### Procedure

To overcome the impact of expectation, we used a cover story. Participants were told to communicate non-verbally with a virtual agent to be tested as a component of an intelligent system. Thus, the participants were naive to the actual study goal.

**Fig. 9:**
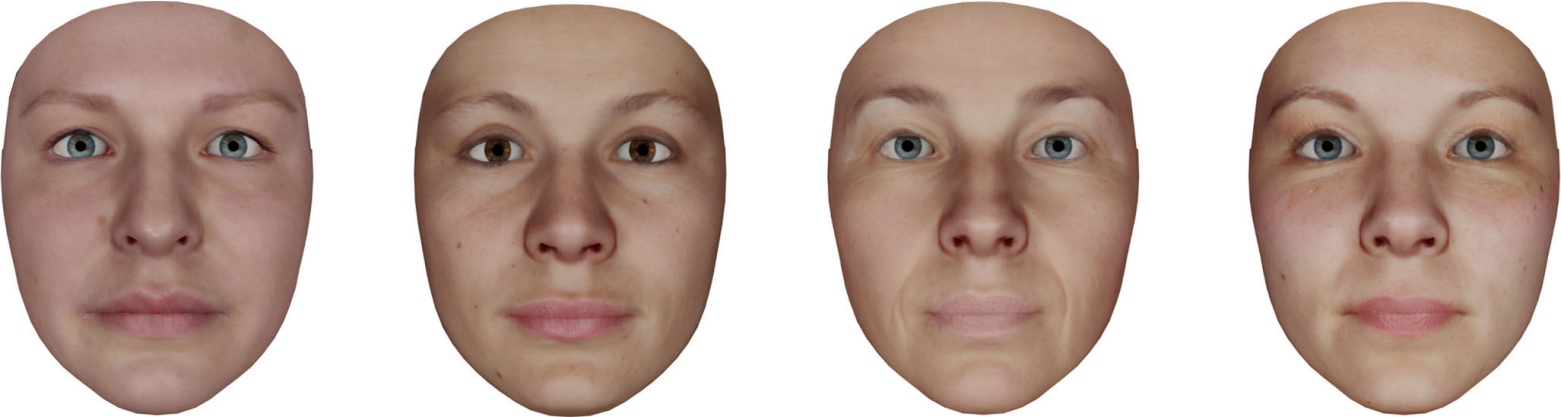
The four female avatars were created for our experiments. We used an average female shape and highly resolved photographic textures of four different individuals contained in the FexMM

The experiment was implemented in PsychoPy. The OVMF was used via the Python interface (see 2). As shown in Fig. 9, we created four female avatars by means of the FexMM for the virtual mirror (Grewe et al., 2021). With this method, we ensured a high semantic congruence of the created avatars.

All individuals were seated in front of a screen and experienced all four conditions within one experimental session, with the four condition blocks being counterbalanced across participants. The baseline condition B was always presented first. There were breaks between the blocks, which lasted 5 min each. To reduce the effect of familiarity with the avatar’s face, we ensured that the avatars were randomly assigned and different for the three subsequent conditions.

Similarly to previous studies (Ma et al., 2016; Ma et al., 2018), the enfacement induction phase lasted 4 min and included three phases. During the first 30 s, participants listened to a recorded verbal instruction about what they were requested to do in the following phases. For the next 180 s, participants were asked to focus on the avatar in the virtual mirror system. The instructions differed depending on the condition.

In condition B, participants were instructed to rotate their heads into different directions and display a few specific facial expressions while they were looking at the avatar. The avatar was animated using the prerecorded rest sequence (see above). In condition P, participants were asked to only rotate their heads and these head movements were imitated by the avatar. Now, the imitating avatar’s expressions always remained neutral. This corresponds to the visuo-motor stimulation that was used in previous enfacement studies (Ma et al., 2018; Serino et al., 2015). In condition E, participants were requested to only display facial expressions. They were imitated by the avatar while its head pose remained at rest. Lastly, in condition PE, participants received the same instruction as in condition B but now with the avatar imitating both, their pose and expressions. At the end of the induction phase, participants were instructed for the remaining 30 s to freely move their head (condition P), display facial expressions (condition E), or do both (conditions B and PE).

Immediately after the induction phase, participants were asked to provide SoA ratings on two different questionnaires. They filled out an established questionnaire (QA1) measuring avatar embodiment with four statements that were assembled from previous studies (Gonzalez-Franco & Peck, 2018). However, this questionnaire was designed for assessing embodiment illusions more generally, and its sensitivity to capture the enfacement illusion has been critically discussed because the face is a more distinctive feature of the self as compared with other body parts (Porciello et al., 2018). Thus, we additionally asked participants to fill out a further questionnaire newly developed for this study (QA2). The questionnaire includes four statements adopted from previous studies on the malleability of facial self-representations (see the supplementary materials). Both questionnaires were presented item by item in a randomized order. Participants indicated their agreement with the statements on a visual analogue scale, ranging from 0 (completely disagree) to 100 (completely agree).

### Results

Because a simple average or sum score of items is not an appropriate indicator for a construct such as SoA (Moreau & Wiebels, 2021), we follow the recommendation of Gonzalez et al. (Gonzalez-Franco & Peck, 2018) and computed a weighted sum score of all items for both questionnaires separately. The basic idea of this approach is to create the sum score by multiplying the row scores of each item with the loading before summation. Thus, according to the magnitude of its association with a latent SoA variable indicated by the loading matrix, every item of the questionnaire is taken to differentially contribute to the sum score (McNeish & Wolf, 2020). To obtain loadings of each item onto the latent SoA variable, we conducted a principal component analysis (PCA) extracting one principal component as a proxy for the latent SoA variable and used the factor loadings provided by this unidimensional PCA. Note that an item’s loading might vary across experimental conditions, leading to biased comparisons between conditions. To alleviate this potential bias, we applied a condition-general PCA simultaneously on the data points collected in all conditions and conducted a measurement invariance test based on factor congruence estimates between the condition-general and condition-specific loadings (using the data points only in one specific condition) afterwards (Fischer & Karl, 2019).

Results indicated measurement invariance with factor congruence coefficients ranging between .84 and .99 for QA1 and between .97 and .99 for QA2. Thus, condition-general loadings of each item can be used for computing the weighted sum score as quantitative indicator of SoA for the upcoming analysis.

Because traditional frequentists’ inference tests only allow categorical decisions about the acceptance or rejection of a null hypothesis (*H*_0_) (Fisher, 1992; Neyman & Pearson, 1933), we additionally report Bayesian statistical indices to signify the relative evidence for or against *H*_0_ (Dienes, 2016). All statistical analyses were performed in R 3.5.1 with a significance threshold of *α* = .05 and a Cauchy prior width of .707 (Morey & Rouder, 2011).

Frequentist and Bayesian inference was applied within exactly the same models, the first generating *p* values for testing the parameter estimates against zero and the second providing Bayes factors (*BF*_10_). *BF*_10_ indicates the relative evidence for the alternative hypothesis (*H*_1_) over *H*_0_. A *BF*_10_ > 1 indicates more evidence for *H*_1_ over *H*_0_ while *BF*_10_ < 1 indicates the opposite. For interpretation, the following semantic labels are usually assigned to the numerical *BF*_10_ values to indicate the magnitude of evidence for *H*_1_ (Jarosz & Wiley, 2014): anecdotal (*BF*_10_ range 1 — 3), substantial (3 — 10), strong (10 — 30), very strong (30 – 100) and decisive (> 100).

#### Results for QA1

The data accumulated with the first questionnaire and aggregated across items followed a normal distribution (multivariate normality test based on skewness: *p* = .162, and kurtosis, *p* = .303). Thus, a one-way repeated measures ANOVA was conducted to compare differences of the weighted SoA sum scores for QA1 across experimental conditions. Mauchly’s test of sphericity indicated that the assumption of sphericity was violated (*χ*^2^(5) = 13.24,*p* = .021). Therefore, a Greenhouse–Geisser correction was applied.

**Fig. 10:**
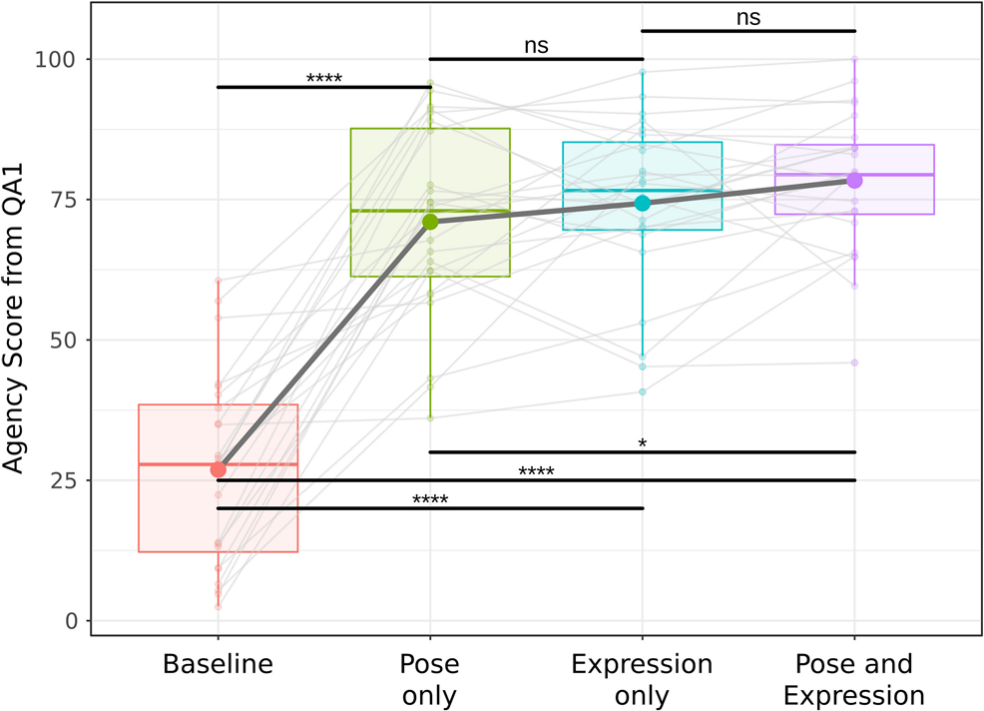
Boxplot of the weighted SoA sum scores assessed with a previously published questionnaire (Gonzalez-Franco & Peck, 2018) (QA1) across the different experimental conditions. The *boxes* denote the interquartile range and the median. Level of significance is indicated by * (*p* < .05), ** (*p* < .01), and **** (*p* < .001)

As shown in Fig. 10, the results revealed a significant main effect of condition on the SoA score (*F*(2.13,49.07) = 94.18,*p* < .001;*η*^2^ = .80). The *BF*_10_ further indicated that the data was at least 1000 times more likely under the model that included the experimental condition as predictor, compared with *H*_0_ of no effect. The evidence was decisive (*BF*_10_ > 1000).

Post hoc tests using the Holm’s correction revealed that the baseline condition B elicited a lower SoA score as compared with the other three synchronous conditions (B: *M* = 27.14,*SD* = 17.19; P: *M* = 71.27,*SD* = 17.06; E: *M* = 74.58,*SD* = 15.20; PE: *M* = 78.64,*SD* = 12.45). All differences were statistically significant (*p* < .001,*p* < .001,*p* < .001, respectively). Bayesian post hoc tests with adjusted prior probability (Westfall, Johnson, & Utts, 1997) of these three comparisons revealed posterior odds which were all larger than 1000, providing decisive evidence for *H*_1_ over *H*_0_, thus in the favor of the alternative, implying condition differences.

Notably, we observed a significantly higher score for the PE as compared to the P condition (*p* = .018). For this comparison, the *BF*_10_ revealed a substantial evidence in favor of *H*_1_ (*BF*_10_ = 3.06), indicating also that the SoA is increased when the avatar’s facial expressions were synchronized in comparison to the pose only condition. However, there was no significant difference between E and PE (*p* = .248). The *BF*_10_ indicated that the data was 3.72 times more likely in the favor of *H*_0_ as compared with *H*_1_. This provides substantial evidence that there is no difference (*BF*_10_ = .27). Finally, there was also no significant difference in SoA between P and E (*p* = .295). The *BF*_10_ indicated substantial evidence to support the *H*_0_, thus revealing no difference between these two conditions (*BF*_10_ = .15).

#### Results for QA2

Analogously to the above, a one-way repeated measures ANOVA was applied for QA2 to compare the weighted SoA sum scores across conditions. The assumption of normality was satisfied (skewness *p* = .141; kurtosis *p* = .974). However, the assumption of sphericity was violated (*χ*^2^(5) = 17.94,*p* = .003), such that Greenhouse–Geisser correction was applied.

**Fig. 11:**
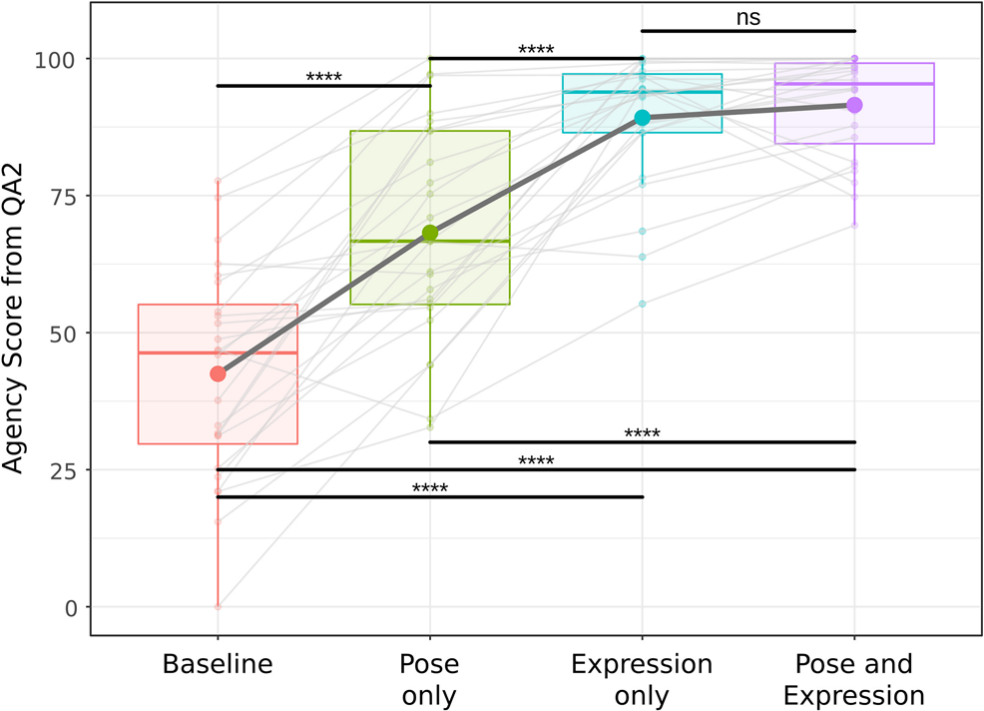
Boxplot of the weighted SoA sum scores assessed by the newly developed questionnaire (QA2) across the different experimental conditions. The *boxes* denote the interquartile range and the median. Level of significance is indicated by * (*p* < .05), ** (*p* < .01), and **** (*p* < .001)

The results for QA2 are shown in Fig. 11. There was a significant main effect of the experimental condition (*F*(2.20,50.63) = 71.96,*p* < .001;*η*^2^ = .76). A decisive evidence supported the inclusion of the experimental condition to explain differences in SoA (*BF*_10_ > 1000).

Follow-up paired samples *t* tests with Holm’s correction indicated that the baseline condition elicited a lower SoA as compared with the other three synchronous conditions (B: *M* = 42.48,*SD* = 19.64; P: *M* = 68.22,*SD* = 19.89; E: *M* = 89.21,*SD* = 12.21; PE: *M* = 91.52,*SD* = 9.52). These differences were all statistically significant (*p* < .001,*p* < .001,*p* < .001, respectively).

A significant difference between the conditions P and PE was indicated by QA2 (*p* < .001). After the adjustment of the prior probability, all *BF*_10_ showed more than 1000 times larger evidence in favor of *H*_1_ for all four comparisons, and thus decisive evidence. In contrast to QA1, we additionally found a significant difference between the P and E condition (*p* < .001). The *BF*_10_ revealed decisive evidence in favor of the *H*_1_ (*BF*_10_ > 1000). There was again no significant difference between E and PE (*p* = .546). Similarly, *H*_0_ was supported by the *BF*_10_ (*BF*_10_ = .17), i.e., no difference between these two conditions.

### Discussion

To evaluate the OVMF for enfacement experiments, we implemented different conditions of facial synchrony between study participants and an imitating avatar, i.e., pose only (P), expression only (E), and pose and expressions (PE). These experimental conditions were compared against a baseline condition (B) with asynchronous subtle movements. As expected, we observed differences in SoA for B as compared with all synchronous conditions. There was a consistently higher experience of agency towards a synchronous avatar, no matter which motion components were transferred. These findings are in line with the previous literature demonstrating congruent motor and visual signals to evoke a feeling of control (Braun et al., 2018). We thus replicate the results of previous enfacement studies that apply visuo-motor stimulation by synchronizing head pose only also with our OVMF (Estudillo & Bindemann, 2016; Serino et al., 2015; Ma et al., 2018). This confirms that it is an effective method to induce enfacement illusions similarly to visuo-tactile stimulation.

We further aimed to disentangle the relative effects of synchronous imitation of head pose and facial expressions. There was a stronger experience of SoA when facial expressions were imitated by the avatar (E) as compared to pose only (P). This finding is consistent with the argument emphasizing a specific importance of non-verbal cues in human communication (Mueser, Grau, Sussman, & Rosen, 1984). A recent study that showed facial expressions to be more impactful than the body in an avatar-mediated virtual environment came to a similar conclusion (Kruzic, Kruzic, Herrera, & Bailenson, 2020). However, our study only revealed significant differences between the P and E conditions on the basis of QA2. Similarly, Kokkinara and McDonnell found maximal ratings of agency but no difference when either pose or expressions were imitated (Kokkinara & McDonnell, 2015). One potential explanation for the effect not being replicable across SoA measurement instruments is that pose and expressions simply contribute equally to the agency experience. Another explanation, however, is that QA1 was designed to assess perceived agency for embodiment more generally (Gonzalez-Franco & Peck, 2018). Consequently, such instruments might be less sensitive in the domain of faces as compared to QA2 which was specifically tailored to the measurement of the enfacement illusion. To investigate the contribution of each motion component, further studies are desirable which also complement self-reports with implicit measures of SoA (Braun et al., 2018).

As expected, we found the strongest SoA experiences in the PE condition, which robustly indicates significant differences as compared with P across questionnaires. This complements the work of Gonzalez et al. (Gonzalez-Franco et al., 2020), suggesting that avatars whose pose is physically synchronized with and whose expressions are animated correspondingly to the participant enhance the enfacement illusion. Our findings thus indicate that the imitation of facial expressions by an avatar constitutes a powerful tool to extend the repertoire of visuo-motor stimulations, and that a combined synchronization of pose and expressions is indeed beneficial for enfacement studies. Virtual mirrors created with the OVMF thus provide intriguing new possibilities for future research.

## General discussion

In this paper, we demonstrated and evaluated the OVMF, an open-source framework for virtual mirror experiments, which is applicable on standard computer hardware. With this framework, psychologists can easily set up enfacement experiments with marker-less tracking of facial pose and expressions.

As discussed in the Introduction to this work, temporal congruence plays a crucial role for the experience of agency. We demonstrated automatized methods for the calibration of our framework’s latency. Our experiments revealed that it can achieve an end-to-end latency that stays fairly below the critical threshold to maintain the experience of SoA. Furthermore, we conducted an experiment that demonstrates the efficiency of our framework to induce the experience of SoA towards an avatar. As such, it might become a more powerful tool than the visuo-motor stimulation in previous experiments, which relied on synchronized head pose only. Our findings are in line with the expectation that facial avatars with additionally synchronized facial expressions strengthen the SoA.

### Advantages of the OVMF

A major advantage of the OVMF is the possibility to achieve strong visuo-motor stimulation in an enfacement experiment which also increases the ecological validity as compared to previous virtual mirrors. Given the ability of very fast markerless face tracking and rendering, a new generation of experiments in enfacement research can be envisioned with the OVMF. For instance, in a conceivable study design, participants could experience the enfacement illusion in a real-time computer-mediated communication context such as in a face-to-face video conference to explore how the plasticity of the self may implicitly change the communicative behavior of individuals. This is facilitated by the modular design of the OVMF that allows easy extension of the framework’s functionality. For instance, the development of new technological modules is possible without knowledge of the framework’s details. In other words, one could easily create and integrate a module to implement traditional visuo-tactile stimulations to compare different approaches of enfacement induction and study their mechanisms. Furthermore, proprietary software for markerless face tracking (e.g., Apple’s FaceKit) or real-time rendering engines (e.g., Unity, Unreal) might be integrated. This also facilitates the usage of immersive display techniques like head-mounted displays in combination with specialized tracking techniques (Thies et al., 2018).

Nevertheless, the current implementation of our framework is fully based on open-source components and provides researchers a sufficient performance using consumer-grade hardware. A comparable performance was hitherto only achieved with expensive professional tracking and display devices. The OVMF also comes with different avatars, which can be animated with a high realism. Despite direct evidence as compared to other avatar setups (Grewe et al., 2021), the successful induction of the SoA in our experiment provides indirect support for a high semantic congruence of the avatars in the context of enfacement.

The OVMF is implemented in Python and seamlessly integrates with other toolboxes, such as PsychoPy, one of the most widely used open experiment control framework in psychology (Peirce et al., 2019). In fact, a growing programming burden for the implementation of experiments which are supported by modern digital technologies such as virtual mirrors exists. This often discourages psychologists from their usage (Cohen, MacWhinney, Flatt, & Provost, 1993). A toolbox that can be easily used with low learning cost is thus arguably of great benefit for the community. With the OVMF, researchers can create and manipulate an enfacement experiment with minimal coding effort in a programming environment which they are familiar with. This can empower more researchers to harness this technology.

Last but not least, the OVMF might serve as a standardized framework. A recent review criticized that the implicit methodological variance of virtual enfacement experiments might be one reason for contradicting findings (Riemer et al., 2019). With our open-source framework, previous experiments can be adapted such that the adjustment of new parameters becomes easier. This fosters transparency at every step of the experimental procedure and data processing such that replications with a standardized and well-controllable method are possible.

### Limitations of our enfacement study

We used subjective ratings on statements which capture different aspects of the SoA in our enfacement experiment. While being the quasi consensus in the field (Kalckert & Ehrsson, 2014b), such ratings were also criticized to be less sensitive to the plasticity of the self-face-representation (Porciello et al., 2018). In future studies, subjective ratings should therefore be complemented with implicit measures, such as an intentional binding tasks or physiological responses. This helps to accumulate broader evidence on the induction of SoA in enfacement experiments (Braun et al., 2018). For instance, a module could be integrated into OVMF, which allows recording of physiological measurements.

In this study, we focused on the SoA, since it is primarily linked to visuo-motor stimulation. Previous works also studied the experience of ownership over an avatar. A future experiment with the OVMF could therefore compare the SoA and the SoO. While we predict a similar increase in measurements of the SoO, this may help to further investigate the co-occurrence of both senses in the enfacement phenomenon.

Only female Caucasian participants were recruited for our study. This potentially limits its generalizability. A future replication could collect a validation sample with a diverse distribution of gender and ethnicity. We expect the OVMF to perform well for a wide variety of participants and conditions, which is made possible by combining robust face tracking via OpenFace with a diversity of avatars that can be created with the FexMM.

### Application of the OVMF beyond enfacement

Besides our goal to develop a new virtual mirror framework for enfacement research, the OVMF was also designed with a broader intention. For instance, it could serve as a tool for researchers which require face tracking to create immediate emotional responses of an avatar. Research designs targeting social interaction in second-person neuroscience might benefit from the ability to control highly realistic face stimuli (Redcay & Schilbach, 2019). Further, the OVMF not only allows real-time control over facial movements but also facilitates on-the-fly manipulation of facial identity parameters. This could help researchers in psychology and neuroscience to study facial mimicry or emotional contagion (Hess, 2021), where the specific manipulation of facial features in video-based stimuli is difficult.

## Data Availability

The OVMF framework is publicly available at https://github.com/mgrewe/ovmf.

## Footnotes

1 https://github.com/mgrewe/ovmf

2 https://facegen.com/

3 https://github.com/animate1978/MB-Lab

4 https://www.michaelgilbert.fr/facshuman/

5 https://github.com/fsotoc/FaReT

6 https://www.psychopy.org/

7 https://osdoc.cogsci.nl/

8 https://opencv.org/

9 https://github.com/TadasBaltrusaitis/OpenFace/

10 https://unity.com/

11 https://www.worldviz.com/vizard-virtual-reality-software

12 https://www.blender.org/

13 https://www.psychologie.hhu.de/arbeitsgruppen/allgemeine-psychologie-und-arbeitspsychologie/gpower
